# Shiga Toxin Increases Formation of Clathrin-Coated Pits through Syk Kinase

**DOI:** 10.1371/journal.pone.0010944

**Published:** 2010-07-27

**Authors:** Audrun Utskarpen, Ramiro Massol, Bo van Deurs, Silje Ugland Lauvrak, Tomas Kirchhausen, Kirsten Sandvig

**Affiliations:** 1 Centre for Cancer Biomedicine, Faculty Division the Norwegian Radium Hospital, University of Oslo, Oslo, Norway; 2 Department of Biochemistry, Institute for Cancer Research, the Norwegian Radium Hospital, Oslo University Hospital, Oslo, Norway; 3 Department of Molecular Biosciences, University of Oslo, Oslo, Norway; 4 Children's Hospital-Boston and Department of Pediatrics, Harvard Medical School, Boston, Massachusetts, United States of America; 5 Department of Cellular and Molecular Medicine, University of Copenhagen, the Panum Institute, Copenhagen, Denmark; 6 Department of Cell Biology and Immune Disease Institute, Harvard Medical School, Boston, Massachusetts, United States of America; University of Birmingham, United Kingdom

## Abstract

Clathrin-dependent endocytosis is a main entry mechanism for the glycolipid-binding Shiga toxin (Stx), although clathrin-independent pathways are also involved. Binding of Stx to its receptor Gb3 not only is essential for Stx retrograde transport to the endoplasmic reticulum and toxicity but also activates signaling through the tyrosine kinase Syk. We previously described that Syk activity is important for Stx entry, but it remained unclear how this kinase modulates endocytosis of Stx. Here we characterized the effects of Stx and Syk on clathrin-coated pit formation. We found that acute treatment with Stx results in an increase in the number of clathrin-coated profiles as determined by electron microscopy and on the number of structures containing the endocytic AP-2 adaptor at the plasma membrane determined by live-cell spinning disk confocal imaging. These responses to Stx require functional Syk activity. We propose that a signaling pathway mediated by Syk and modulated by Stx leads to an increased number of endocytic clathrin-coated structures, thus providing a possible mechanism by which Stx enhances its own endocytosis.

## Introduction

The bacterial toxin Shiga toxin (Stx) binds via its pentamer of B-subunits to the glycosphingolipid receptor Gb3 on the cell surface and is endocytosed both by clathrin-dependent and clathrin-independent mechanisms (reviewed in [1]). The toxin then follows the retrograde pathway to the endoplasmic reticulum [2] and is translocated to the cytosol, where the A-subunit inhibits protein synthesis. Stx is internalized partially via clathrin-coated pits, and the clathrin-dependent uptake increases with higher concentrations of Stx [3], [4]. Upon binding to its receptor, Stx activates signaling cascades leading to apoptosis, as well as more rapid signaling through the Src family kinases Lyn [5] and Yes [6], the MAP kinase p-38 [7], the serine/threonine kinase PKCδ [8], and the tyrosine kinase Syk [9], [10]. We have previously demonstrated that Syk regulates Stx uptake and that Stx induces activation of Syk, which in turn phosphorylates clathrin heavy chain (CHC). In addition, Stx promotes the formation of a complex between clathrin and Syk [9], [10].

There is evidence that some types of cargo that bind to transmembrane protein receptors and are taken up via clathrin-coated pits and vesicles may affect recruitment of clathrin to the plasma membrane. Binding of epidermal growth factor (EGF) to its tyrosine kinase receptor has been reported to increase clathrin phosphorylation through a downstream activation of Src kinase [11] and also to induce the formation of clathrin-coated pits [11], [12]. Similarly, neuronal growth factor (NGF) has been found to augment clathrin phosphorylation and clathrin recruitment to the plasma membrane in neuronal cells, presumably through phosphorylation of its receptor tyrosine kinase TrkA [13], [14]. Although studies in fixed cells indicate that ligands may promote recruitment of clathrin, live-cell imaging is needed to determine if there is indeed an increased formation of endocytic clathrin-coat structures or just an accumulation of coated pits due to altered signaling.

Motivated by our previous findings that Stx can mediate its own uptake and affect signaling, we set out to investigate if Stx, although binding to a glycolipid, was able to increase the formation of endocytic clathrin coat structures at the plasma membrane and if such increase in clathrin coats might be mediated by signaling through the Syk kinase. We used a combination of live-cell spinning disk confocal imaging and electron microscopy to study if and how Stx might affect the formation of clathrin-coated structures. We observed a significant increase in the number of clathrin-coated pits upon incubation with Stx, and their lifetime and intensity distributions were shifted towards higher values. Presence of the Syk inhibitor piceatannol prevented the formation of new clathrin-coated structures induced by StxB, indicating a role for Syk in the formation of clathrin-coated pits. Taken together our data suggest that Stx can increase the efficiency of clathrin-coated pit formation through a Syk-dependent mechanism.

## Results

### Stx and StxB increase recruitment of clathrin and AP-2 to the plasma membrane

**Figure 1:**
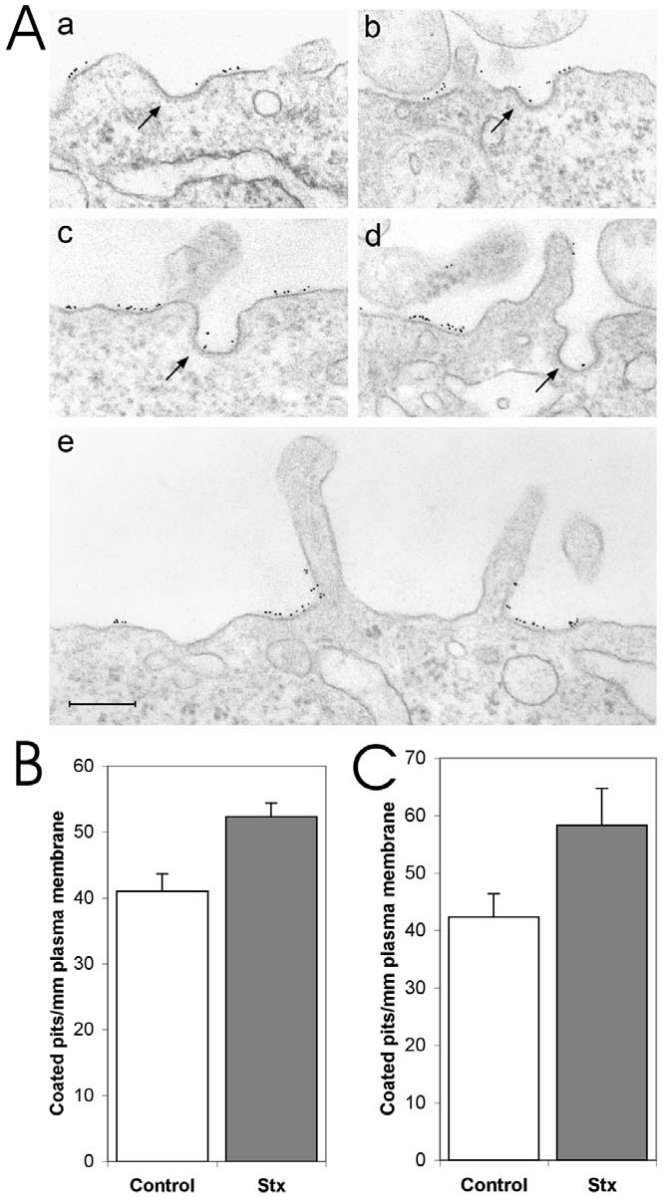
Stx increases formation of clathrin-coated pits. (**A**) Representative electron micrographs of immunogold-labeled HeLa cells showing Stx associated with clathrin-coated profiles (arrows in a–d) as well as associated to non-specialized regions of the plasma membrane (a–e). Bar 200 nm. (**B**) Exposure of HeLa cells to Stx for 10 min increased the number of clathrin-coated pits at the plasma membrane with statistical significance by 28±4% (mean ± SE, p = 0.008, n = 3). (**C**) Exposure of HEp-2 cells to Stx for 10 min resulted in a 38±2% statistically significant increase in the number of clathrin-coated pits at the plasma membrane (mean ± SE, p = 0.004, n = 3). Data were obtained from 20–30 cell profiles, containing from 38 to 63 pits altogether, per experiment and condition. Data are displayed in the plots as mean ± SD.

Based on the previous findings that Stx can induce signaling in cells and promote its own uptake through a clathrin-mediated pathway, we investigated if Stx was able to influence the formation of clathrin-coated pits and vesicles. Electron microscopy was used to quantify the number of clathrin-coated pits per mm of plasma membrane before and after incubating cells with Stx for 10 min at 37°C. Both HeLa and HEp-2 cells were tested, and we observed an increase in the number of clathrin-coated profiles upon Stx stimulation of 28±4% and 38±2% (mean ± SE), respectively (fig 1B–C). Electron microscopy of Stx immunogold-labeled HeLa cells reveals that Stx is present in clathrin-coated pits as well as on non-specialized regions of the plasma membrane (fig 1A).

All following experiments were performed in HeLa cells stably expressing the σ2 subunit of the adaptor complex AP-2 fused to EGFP. Like in previous studies, we used AP-2 as a fiduciary for endocytic clathrin-coated structures because AP-2 is exclusively recruited to all clathrin-coated structures forming at the cell surface whereas it does not mark endosomal clathrin-containing structures [15]–[18]. It is also well established that AP-2 recruitment dynamics accurately reflects the properties of endocytic clathrin coats and that the accumulated fluorescence signal within a spot is proportional to the size of the clathrin-coated structure [15], [17]. These experiments were all carried out using StxB subunit (StxB-Sulf2), which is sufficient for both binding to the lipid receptors at the cell surface and for inducing signaling. Similar to what we observed by electron microscopy, we also found an increase in the number of clathrin-coated structures as determined by spinning disk confocal live-cell imaging. First, 3 min control movies on chosen groups of cells were recorded, followed by addition of Alexa Fluor 568-labeled StxB. Time-series (3–10 min) of the same group of cells as used for the control movies were then recorded, starting at different times after the addition of StxB. The number of clathrin-coated structures identified by σ-EGFP AP-2 spots at the bottom surface of each cell in direct contact with the glass coverslip was determined at different time points. StxB internalization was established by the appearance of perinuclear staining of StxB-Alexa Fluor 568 following 20–30 min of incubation (data not shown). Only cells found to internalize StxB were included in the analysis.

**Figure 2:**
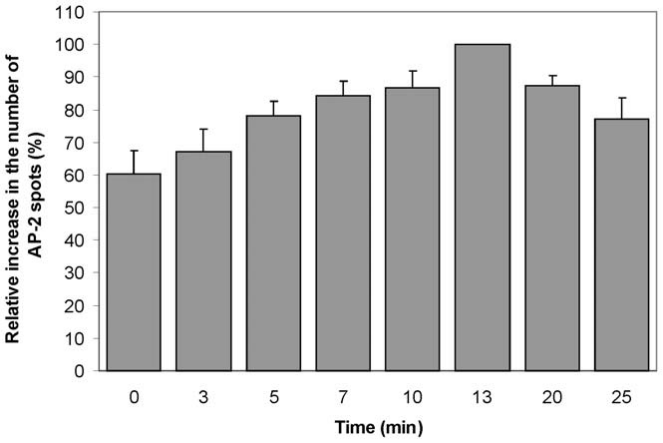
The number of AP-2-containing structures increases after incubation with StxB. Time-series obtained by spinning disk confocal microscopy of the ventral surface of HeLa cells stably expressing σ2-EGFP were recorded before and after addition of StxB. The σ2-EGFP positive spots are fiduciaries of endocytic clathrin-coated structures. The number of clathrin-coated structures obtained from 9 independent experiments was determined as described in Materials and Methods. The maximum number of AP-2 spots in each cell was set equal to 100%, and the number of AP-2 spots at other time points was then calculated as fractions of the maximum number. Results are displayed ± SE or average deviation. The raw data for each cell are displayed in table 1 (cells 1 to 9).

The number of AP-2 spots from 16 independent experiments is shown in table 1. In most cells, there was a rapid increase in the number of endocytic AP-2/clathrin-coated structures within 3–7 min after addition of StxB, reaching a maximum value after 10–13 min (fig 2 and table 1). These results are in agreement with earlier reports on the fast effect of Stx-induced signaling [9]. Out of 16 cells analyzed, 2 cells had no detectable increase in the number of clathrin-coated pits, 6 cells had a maximum increase of 20–50%, whereas 8 cells showed a maximum increase of 60–190% with respect to their own controls (determined immediately before StxB addition). The mean increase ± SE in the number of clathrin-coated pits/vesicles after 10 min of StxB addition was 57±16% (p = 0.002).

**Table 1:** Number of AP-2 spots in 16 cells before and after incubation with StxB for different periods.

Cell	Control	Number of AP-2 spots at different times after addition of StxB						
	0 min	3 min	5 min	7 min	10 min	13 min	20 min	25 min
1	31	65	64	61	77			79
2	7	19	20	20	19			28
3	62	51	64	72	84			55
4	75	74	72	79	111			71
5	75	46	63	115	79			54
6	90	83	88	96	95			87
7	49		93	107	107	115	97	78
8	54		76	71	84	93	84	75
9	76		98		64			
10	195				302			
11	70				86			
12	59				80			
13	21				33			
14	38					96		
15	61					95		
16	51					56		

Time-series obtained by spinning disk confocal microscopy of the ventral surface of 16 different HeLa cells stably expressing σ2-EGFP were recorded before and after addition of StxB. The σ2-EGFP-containing spots correspond to the recruitment of AP-2 adaptors to the plasma membrane and are considered fiduciaries of endocytic clathrin-coated structures [15], [17], [19]. The numbers of clathrin-coated structures before (control) and at different time points after addition of StxB are shown for 16 independent experiments.

### The new AP-2-containing structures formed upon StxB addition behave like canonical clathrin-coated pits

**Figure 3:**
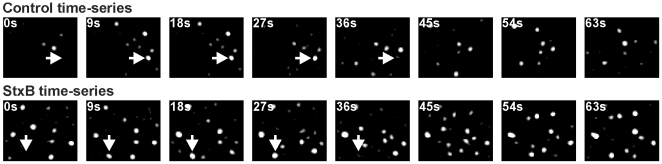
StxB increases the activity of AP-2 structures. Representative time-series were obtained by live-cell spinning disk confocal imaging of a HeLa cell stably expressing σ2-EGFP before (upper panel) and after (lower panel) exposure to StxB for 10 min. Arrows point to examples of diffraction-limited AP-2 spots appearing and disappearing within the time-series. Images were acquired every 3 s, and are shown with 9 s between images. Laplacian 2D and Gaussian filtering were applied on all images in order to facilitate their graphical display. The time series corresponding to this experiment is available as Supporting Information (Movie S1).

To further understand the behavior of AP-2/clathrin-coated structures in cells exposed to StxB, we followed the kinetics of AP-2 recruitment in living cells with time series acquired using spinning disk confocal microscopy. Representative examples obtained towards the periphery of a HeLa cell before and after addition of StxB are presented in fig 3 (white spots; arrow heads highlight selected examples) and Movie S1 (green and red correspond to the AP-2 spots in the same cell before and after addition of StxB). There was an increase in the number of AP-2 structures detected after StxB treatment. In general they had a lifetime in the range between 30 and 90 s, and their fluorescence intensity increased gradually as expected for the behavior of canonical endocytic clathrin-coated pits in HeLa cells [17]. Significantly longer-lived AP-2 structures (lasting more than 2 min) corresponding to coated plaques were not included in the analysis. These structures were relatively sparse in the region of observation located towards the periphery of the plasma membrane of the HeLa cells attached to the glass coverslip.

### Lifetime is increased and maximum fluorescence intensity distributions of AP-2 are shifted upon StxB stimulation

**Figure 4:**
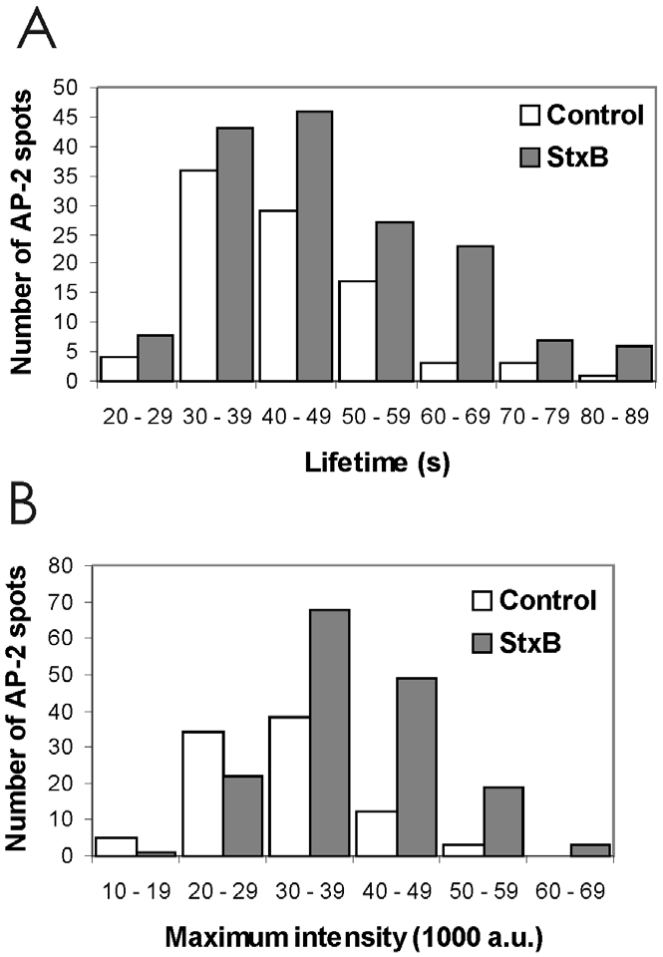
Lifetime and maximum intensity distributions of AP-2 spots are shifted upon StxB treatment. Representative results obtained from a HeLa cell stably expressing σ2-EGFP are shown. A control time-lapse series was first recorded for 3 min, and then another 3 min time-lapse series was acquired after 10 min of incubation with StxB. Images were acquired every 3 s. A total of 92 (control) and 162 (after StxB-treatment) AP-2 spots were recorded and analyzed. Only spots appearing and disappearing within the time-lapse period of 3 min and typically lasting between 7 and 30 timeframes (21 to 90 s) were included in the analysis. (**A**) Effect of StxB on the lifetime distribution of the AP-2 spots. White and grey bars represent the number of AP-2 clusters in the different lifetime intervals of the histogram for the control and the StxB-data set, respectively. Lifetime was defined as the period from appearance to disappearance of the AP-2 spot. (**B**) Effect of StxB on the distribution of maximum fluorescence intensity for AP-2 spots. White and grey bars represent the number of AP-2 spots in the different maximum intensity intervals of the histogram for the control and the StxB data set, respectively. Maximum fluorescence intensity is expressed in arbitrary fluorescence units and was measured just prior to dissolution of the AP-2 signal due to un-coating of the clathrin/AP-2 coat.

The effect of StxB on the lifetime and maximum fluorescence intensity distributions of the canonical clathrin-coated pits was determined using an image analysis package (IMAB) developed in MATLAB [19]. In three independent experiments, time series of 3 min in duration were obtained in the same cells before and after 10 min incubation with StxB. Depending on the time series and the region covered for observation, we detected anywhere between 50 and 162 AP-2 spots whose behavior correspond to the criterion of canonical clathrin-coated pits [15], [18]. The data presented in fig 4 displays a representative outcome from one such experiment. As expected, there was an increase in the overall number of AP-2 spots observed upon StxB incubation (74% increase) and most of the new ones had longer lifetimes (fig 4A; mean lifetime ± SD of 43±11 s before and 49±14 s after addition of StxB). It has been demonstrated that longer lifetimes correlate well with larger maximum intensities [15]. Indeed, incubation with StxB also leads to an increase in the maximum fluorescence intensity of the AP-2 spots (fig 4B). Similar results were observed in the other cells analyzed.

### StxB-incubation does not alter the extent of transferrin endocytosis

**Figure 5:**
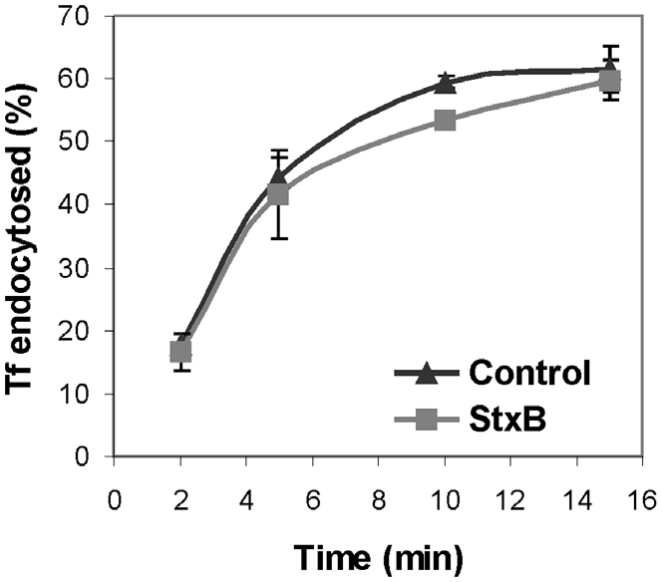
Rate of Tf internalization in HeLa cells is not affected by the addition of StxB. At different time points ^125^I-labeled Tf was added to all wells in the presence or absence of StxB. The ^125^I-signals corresponding to endocytosed and surface bound fractions were determined. Data are expressed as the mean ± average deviation of results from two independent experiments done in duplicate.

Since transferrin (Tf) is internalized mainly via clathrin-dependent endocytosis, we tested whether the extent of Tf uptake was affected upon treatment of HeLa cells with StxB. We found no significant increase in the extent of Tf uptake in two independent experiments done by following the internalization of ^125^I-Tf for 2, 5, 10, and 15 min after its addition to the medium (fig 5).

### The increase in the number of AP-2 structures induced upon StxB incubation is prevented by the Syk inhibitor piceatannol

**Figure 6:**
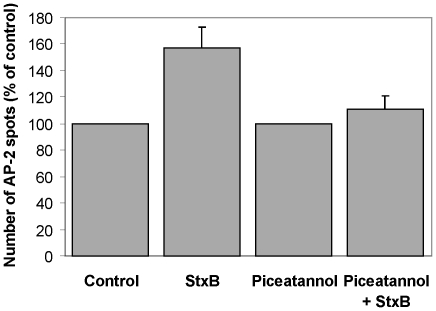
Inhibition of Syk by piceatannol inhibits the Stx-induced increase in the number of AP-2 spots. HeLa cells stably expressing σ2-EGFP were pre-incubated with piceatannol (50 µM) for 30 min. A time-lapse series was acquired by spinning disk confocal microscopy with an image obtained every 3 s for 1–2 min. The cells were then exposed to StxB for 10 min, followed by the acquisition of a second time-lapse series of 1–2 min. Six independent experiments, with 1–4 cells per experiment, were analyzed, and the result is expressed relative to piceatannol only. There was no statistically significant increase (p = 0.2) in the number of AP-2 spots when adding StxB upon piceatannol treatment. For comparison, the number of AP-2 spots before (control) and after 10 min incubation with StxB is shown as determined from table 1; the result is expressed relative to the control and shows a statistically significant increase of 57±16% (mean ± SE, p = 0.002).

We have previously demonstrated that the Syk kinase is activated by Stx/StxB and that this activation is important for Stx uptake. In addition, Stx/StxB stimulate the formation of a complex between Syk and clathrin [9], [10]. These findings led us to investigate if Syk might be important for the increased clathrin/AP-2 recruitment caused by StxB. HeLa cells stably expressing σ2-EGFP were pre-incubated with the Syk inhibitor piceatannol (50 µM) for 30 min followed by the acquisition of a spinning disk confocal time-series of 1–2 min duration. StxB was then added followed by the acquisition of a second time series 10 min thereafter. Six independent experiments were conducted, and the number of AP-2 spots from 1–4 cells shown to internalize StxB was determined (fig 6). In contrast to the stimulation of AP-2 recruitment by StxB alone (fig. 2, table 1) we do not find any significant increase in the number of AP-2 spots in cells first incubated with piceatannol and then with StxB (p = 0.2).

Control experiments indicate that the formation of AP-2 containing structures was not affected by treatment with piceatannol or carrier (DMSO) only. After 30 min of incubation with piceatannol the amount of AP-2 spots relative to the control (100%) was 104±3% (mean ± average deviation, 4 cells analyzed per experiment; two experiments). Likewise, treatment of cells with DMSO for 30 min in 2 independent experiments did not affect the number of AP-2 spots (103±10%; mean ± average deviation) relative to the control (100%).

## Discussion

Stx was the first lipid-binding ligand shown to induce its own uptake through a clathrin dependent pathway [3]. Our data provide evidence that Stx/StxB, upon binding to its glycolipid receptor at the cell surface, is able to increase the formation of endocytic clathrin-coated structures directly detected by electron microscopy and indirectly by the appearance of AP-2 spots at the plasma membrane. Furthermore, data obtained using live cell spinning disk confocal imaging indicates that the new coated structures tend to have longer lifetimes and higher maximum fluorescence intensities, presumably reflecting slightly larger coats. The tyrosine kinase Syk seems to be important for this activation, although the mechanism responsible for this process remains to be established.

We first used electron microscopy to demonstrate an increase in the number of clathrin-coated pits in HeLa or HEp-2 cells exposed to Stx. We then confirmed this finding for HeLa cells exposed to StxB by using live-cell spinning disk confocal imaging. We note that our data might underestimate the full extent of the effect because we did not include in the analysis clathrin/AP-2 coated plaques, corresponding to endocytic structures lasting more than 2–3 min [17]. We noticed differences in the response of given cells that we interpret to be due to the existence of cells that poorly bind Stx, probably because of differences in the expression level of the Stx glycosphingolipid receptor Gb3.

It is presently unclear whether the increase of AP-2 structures mediated by Stx is explained by activation of initiation events leading to an increase in the number of the de novo formed coated pits or whether Stx activation stabilizes abortive pits allowing them to become fully formed coated vesicles [15], [18], [20]–[22]. Stx can induce non-apoptotic signaling cascades in cells, but the mechanism behind this rapid signaling, induced by binding of its B-moiety to Gb3, remains to be established [3]–[10]. It has been proposed that the lipid tail of glycosphingolipids can interact with the inner membrane leaflet, and signaling may also be mediated through a membrane protein interacting with the Stx/Gb3-complex. It has recently been shown that the extra-cellular domain of the trans-membrane receptor Fas interacts strongly with Gb3 through a glycolipid-binding motif [23]. This motif is important for Fas-induced apoptotic and non-apoptotic signaling as well as clathrin-mediated internalization of Fas [23]. Moreover, Stx has been shown to interact with two non-identified, possibly signaling proteins at the cell surface [24]. We have previously demonstrated that increased concentrations of Stx, as opposed to StxB, lead to higher uptake of TAG/biotin-labeled Stx [4]. Thus, the A-moiety seems to be required for the concentration-dependent toxin uptake, yet StxB is sufficient for signaling and increased clathrin-coated pit formation.

Our data show that the Stx-induced formation of the AP-2-containing clathrin-coated structures reached its maximum value after about 10–13 min of incubation with the toxin. This observation coincides with the timing of clathrin heavy chain phosphorylation induced by Stx that peaks at around 10–15 min after toxin addition [9]. Of note, activation of Syk by Stx is faster and can be detected already 2.5 min after addition of Stx [9]. Clathrin and Syk can associate with each other, and they form a complex upon induction by Stx [10]. Syk is not only important for Stx endocytosis [9], [10] but it also seems to be involved in the uptake of human rhinovirus [25]; interestingly, virus binding induces Syk recruitment to the plasma membrane and co-association with clathrin. We found that inhibition of Syk activity by piceatannol prevented the increase in the number of clathrin-coated structures that formed upon addition of StxB. Although these observations clearly indicate a connection between Syk activation and regulation of the clathrin endocytic pathway, the detailed mechanism responsible for this process remains to be determined. These observations also seem to rule out a simple model of clathrin coat activation that could be based on changes in the physical properties of the lipid bilayer at the site of coat formation due to local clustering of glycosphingolipids induced by binding to the pentameric StxB.

## Materials and Methods

### Materials

Stx was provided by JE Brown (USAMRIID, Fort Detrick, MD) and StxB (StxB-Sulf2) was prepared as described in [26]. StxB was labeled using an Alexa Fluor 568 protein labeling kit from Invitrogen (Carlsbad, CA) and purified on an Illustra NAP-5 Column (GE Health Care, Waukesha, WI) following the manufacturers' instructions. Degree of labeling was calculated to 3 mole dye per mole protein. Horse anti-Stx serum was obtained from Bureau of Biologics, Food and Drug Administration (Bethesda, MD). Piceatannol came from BIOMOL (Plymouth Meeting, PA) and was re-suspended in DMSO. DMSO concentrations in experiments never exceeded 0.2%. All other chemicals were from Sigma-Aldrich (St. Louis, MO) unless otherwise stated.

### Cell culture

All cells were cultured in DMEM supplied with 10% fetal calf serum, 2 mM L-glutamine, penicillin (50 units/ml), and streptomycin (50 mg/ml) at 37°C and 5% CO_2_. For live imaging experiments HeLa cells stably expressing σ2-EGFP [27] were seeded at a density of 10^5^ cells on glass cover-slips 25 mm in diameter (No. 1.5, Warner Instruments, Hamden, CT) in 6-well plates two days prior to the experiment. For electron microscopy HeLa and HEp-2 cells were seeded the day before the experiment at a density of 4×10^5^ cells per 25 cm^2^.

### Electron Microscopy

Two types of ultrastructural experiments were performed. The cells were starved in MEM supplemented by Hepes at 37°C for 4 h. In the first experiment HeLa and HEp-2 control cells and cells exposed to Stx (250 ng/ml for 10 min at 37°C) were washed 3 times in cold buffer (0.14 M NaCl, 2 mM CaCl_2_, 20 mM Hepes, pH 7.0), fixed (0.1 M cacodylate buffer, pH 7.2, and 2% glutaraldehyde), dehydrated, and embedded in Epon. Sections from the four samples were examined by EM without knowing the specific experimental setup, and 20–30 cell profiles from each experiment were selected at random and photographed. The length of the plasma membrane was measured on these images. In addition, the number of CCPs at the plasma membrane of the same cell profiles was counted at high magnification in the microscope. In the other experiment, HeLa cells were incubated with Stx (10 µg/ml) for 10 min at 37°C, and after washing further incubated with anti-Stx antibody for 60 min on ice. Thereafter the cells were incubated with protein G-gold (PGG) on ice (60 min), fixed and further processed for EM (pre-immunogold labeling, see [28]).

### Live-cell imaging

HeLa cells stably expressing σ2-EGFP were seeded 2 days prior to the experiment. The cells were serum-starved for 1½–2 h in Hepes medium. The cover-slip was transferred to a chamber insert (20/20 Technology, Wilmington, NC) containing Hank's balanced salt solution (HBSS) supplemented by 15 mM Hepes, 4.5 g/L glucose, and 350 mg/mL Na_2_HCO_3_, and pH-adjusted to 7.6, and the cells were maintained at 37°C and 5% CO_2_ throughout the experiment. A Mariana^tm^ system (Intelligent Imaging Innovations, Denver, CO) consisting of an Axiovert 200M epifluorescence microscope (Carl Zeiss, Thornwood, NY) with a spinning disk confocal head (CUX-22, Yokagawa, Japan) was used for spinning disk confocal imaging. It was also equipped with a motorized spherical aberration correction unit (Intelligent Imaging Innovations, Denver, CO). Solid state 473 and 561 nm lasers were used to excite EGFP or Alexa Fluor 568; the objective was a 63× Plan Apo DIC with numerical aperture 1.40 (Carl Zeiss, Thornwood, NY), and the camera was a CCD Cascade II (Photometrics, CA). The microscope was controlled by SlideBook 4.2 software (Intelligent Imaging Innovations, Denver, CO). A typical experiment started with the acquisition of control movies of 3 min recorded from the plasma membrane at the bottom of the cell in contact with the glass coverslip, with 30 ms exposure every 3 s. Then StxB-Alexa Fluor 568 (final concentration 5 µg/ml) plus StxB (final concentration 0.2–2 µg/ml) were added. New movies were captured of the same areas in the same cells, starting 3–8 min after addition of StxB. After 30 min the medium was changed to fresh HBSS, and a 3D-stack was acquired to test toxin uptake as reflected by the extent of perinuclear StxB staining. For the experiments including piceatannol, the cells were incubated in HBSS containing 50 µM piceatannol for 30 min before control movies were acquired, and piceatannol was present throughout the experiment.

### Image processing

Images were processed using SlideBook 4.2; the number of AP-2 structures (equivalent to endocytic clathrin-coated pits and vesicles) observed at given time points was determined as follows: The sum of 20 timeframes corresponding to a 1 min movie starting at a specific time point was projected as a single image, and the total number of AP-2 spots present in the time series before and after addition of StxB was counted. Dynamics of coated pit formation was determined with the aid of an image analysis application (IMAB) for MATLAB 7 (Mathworks, Natick, MA) as described in [19].

### Tf uptake

HeLa cells were seeded in 6-well plates at a density of 2×10^5^ one day prior to the experiment. They were serum-starved for 2 hours in Hepes medium, and ^125^I-labeled Tf was added to all cells with or without StxB (StxB-Alexa Fluor 568 (5µg/ml) plus unlabeled StxB (2 µg/ml)) for the indicated times at 37°C. The cells were then washed three times in Hepes buffer (0.14 M NaCl, 2 mM CaCl_2_, 20 mM Hepes, pH 7,2) at 4°C, followed by addition of Pronase (Roche, Mannheim, Germany) in Hepes buffer. After 1 h incubation on ice, cells were centrifuged, and radioactivity in the pellet and in the supernatant was counted with an LKB 1261 Multigamma counter (Wallac, Turku, Finland).

#### Statistical analysis

The paired Student's *t*-test was used to determine the level of statistical difference between the means of two groups of data. Minimum level of significance was set at p = 0.05.

## Supporting Information

Movie S1 Live-cell spinning-disk confocal imaging of a HeLa cell stably expressing σ2-EGFP before and after addition of StxB. The composite movie overlays two consecutive 3 min time-lapse series acquired from the same cell area before (green) and after (red) incubation for 10 min with StxB. The arrow points to a representative diffraction limited AP-2 spot appearing and disappearing within the time-lapse. Laplacian 2D and Gaussian filtering were applied to the time-lapse series to facilitate the graphic visualization [15] The last part of the movie compares the location at which new pits formed during the time series before and after addition of StxB.
(1.79 MB MOV)
